# Correction: Alfei, S.; Zuccari, G. Recommendations to Synthetize Old and New β-Lactamases Inhibitors: A Review to Encourage Further Production. *Pharmaceuticals* 2022, *15*, 384

**DOI:** 10.3390/ph15050526

**Published:** 2022-04-25

**Authors:** Silvana Alfei, Guendalina Zuccari

**Affiliations:** Department of Pharmacy (DIFAR), University of Genoa, Viale Cembrano, 16148 Genoa, Italy; zuccari@difar.unige.it

## Error in Table

In the original publication [1], there were two mistakes in **Table 3** as published. **In the chemical structure of ANT-3310 (column 2, row 16) the NO bond was not clearly visible, and in the chemical structure of ANT-2681 (column 2, row 22), an amide functionality, was linked to the thiazole ring in place of an acid one.** The corrected **Structures of ANT-3310 and ANT-2681** appear below.


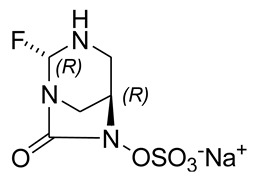


ANT-3310


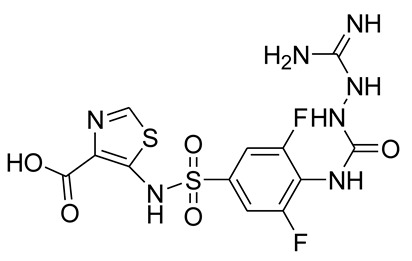


ANT-2681

## Error in Schemes

In the original publication, there was a mistake in **Scheme 16** as published. **In the first three chemical structures of Scheme 16, a BnO ester functionality was reported in the place of an EtO one.** The corrected **Scheme 16** appears below.

In the original publication, there was a mistake in **Scheme 23** as published. **In the final structure of ANT-2681, the PMB protecting group should not have been present.** The corrected **Scheme 23** appears below.

The authors apologize for any inconvenience caused and state that the scientific conclusions are unaffected. The original publication has also been updated.

## Figures and Tables

**Scheme 16:**
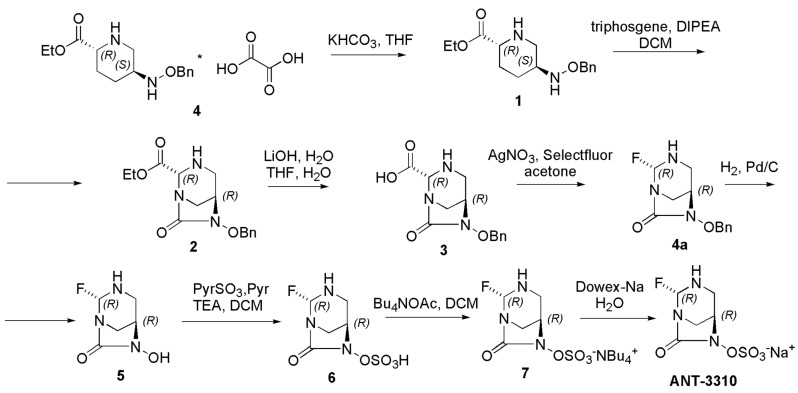
Synthesis of ANT-3310 (C_6_H_9_ FN_2_O_5_S, MW = 240.2), IUPAC name, (2R,5R)-2-fluoro-7-oxo-1,6-diazabicyclo [3.2.1] octan-6-yl hydrogen sulphate.

**Scheme 23:**
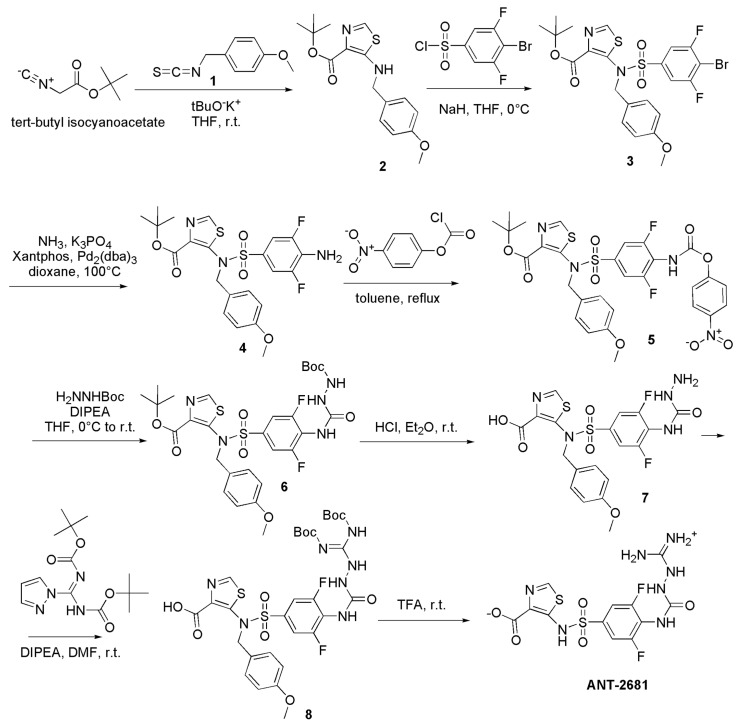
Synthesis of ANT-2681 ANT-2681 (C_12_H_12_F_2_N_8_O_4_S_2_, MW = 434.4), IUPAC name: 5-((4-(2-Carbamimidoylhydrazine-1-carboxamido)-3,5-difluorophenyl) sulphonamide) thiazole-4-carboxamide.
